# TNF-α Promotes the Recovery of Dorsal Root Ganglion Neurons from Cisplatin-Induced Injury Through an NGF-Independent Mechanism

**DOI:** 10.3390/cimb47070482

**Published:** 2025-06-24

**Authors:** Yiling Wei, Xianlin Xu, Pan Wu, Xiang Chen, Qingmei Mo, Ming Zhuo

**Affiliations:** 1 Medical School, Guizhou University, Guiyang 550000, China; yilingweiv@163.com (Y.W.); panwu20011020@163.com (P.W.); xiangchen18@163.com (X.C.); qingmeimo@163.com (Q.M.); 2 Department of Biochemistry, School of Preclinical Medicine, Zunyi Medical University, Zunyi 563100, China; xuxl@zmu.edu.cn

**Keywords:** CIPN, DRG, tumor necrosis factor α, neurite regeneration, DNA damage, mitochondrial damage, TNFR2, nerve growth factor

## Abstract

Nerve injury caused by chemotherapy drugs is a common side effect. How to reduce this kind of nerve injury and promote neuron recovery is of great significance. In this study, we found that tumor necrosis factor-α (TNF-α) promoted the recovery of dorsal root ganglion (DRG) neuron from cisplatin-induced injury. On DRG neurons cultured in vitro, we found that TNF-α promoted neurite regeneration after cisplatin injury. In addition, TNF-α accelerated the removal of DNA damage and promoted the regeneration of mitochondria on DRG neurons. Study of the mechanism showed that this effect of TNF-α was independent from the NGF signaling pathway and occurred mostly through the activation of TNFR2 receptors, together with nucleus translocation of p65 and upregulation of NF-κB expression. This study provides a new theoretical basis and therapeutic strategy for the treatment of nerve injury caused by chemotherapy drugs.

## 1. Introduction

Chemotherapy-induced peripheral neuropathy (CIPN) severely limits the clinical application and dose adjustment of chemotherapy drugs, and may even lead to the discontinuation of chemotherapy [1]. About 50–90% of chemotherapy patients will develop CIPN, and 30–40% of them will progress to chronic neurological adverse reactions [2,3,4]. Dorsal root ganglion (DRG) is a functional center of sensory transduction and regulation, while it is also the main target of chemotherapy drugs. Research findings from various studies suggest that chemotherapy agents can cause direct damages to dorsal root ganglion (DRG) neurons as well as peripheral nerves. These damages may trigger the deterioration of sensory fibers or the depletion of small nerve fibers [5,6]. Additionally, these drugs can impair microtubule integrity within neuronal cells thus disrupting axonal transport mechanisms that rely on microtubules. They may also interfere with mitochondrial activity or induce DNA damage [7], ultimately contributing to the development of peripheral neuropathy or small fiber neuropathy.

Cisplatin is a class–ic chemotherapy drug which can cause severe DRG neuron injury. In DRG neurons, cisplatin can bind to both nuclear DNA and mitochondrial DNA (mtDNA) and induce DNA cross-linking damage, which in turn interferes with the function of DNA. Abnormal gene expression causes extensive damage to both cytoplasm and mitochondria, ultimately triggering apoptosis or other forms of cell death [8].

Nerve growth factor (NGF) is a classic neurotrophic factor widely used in culturing DRG neurons. It can regulate neuronal development, cell death, and survival [9,10]. NGF plays an important role in the axonal regeneration of damaged sensory neurons [11,12]. Tumor necrosis factor alpha (TNF-α) is a pleiotropic cytokine that mediates pro-inflammatory responses and regulates immunity. It is named for its ability to induce tumor cell necrosis. After DRG tissue injury, macrophages and neutrophils rapidly accumulate at the injured site and become activated to produce TNF-α within a relatively short period of time [13]. TNF-α has diverse effects on peripheral nerves. Studies have shown that after sciatic nerve injury, an increase in the number of macrophages and amount of TNF-α in the DRG is observed, and the interaction between TNF-α and neurons enhances the regenerative capacity of neurons and supports neurite outgrowth [14]. However, when exogenous TNF-α is directly injected into the sciatic nerve or DRG of rats, even within the physiological concentration range, it induces thermal hyperalgesia and mechanical allodynia [15,16]. The specific mechanisms underlying these effects are not fully understood, indicating the complexity of TNF-α’s role in the function of the peripheral nervous system [17]. Interestingly, there seems to be an interaction between NGF and TNF-α. It has been reported that TNF-α can synergistically promote the expression of NGF in cells [18,19,20,21], thus promoting the regeneration of injured peripheral nerves.

TNF-α exerts its biological effects through binding to tumor necrosis factor receptor 1 (TNFR1) or tumor necrosis factor receptor 2 (TNFR2). Studies have shown that treatment with TNF-α in hippocampal neurons of TNFR2 knockout mice or TNFR1 overexpression mice resulted in neuronal apoptosis in both cases, indicating that TNFR1 had some kind of neurotoxic effect [22], which shows that the diversity of TNF-α activity mediated by different receptors is an important part for its functional complexity.

In order to understand the effect of TNF-α on DRG neurons, we analyzed its effect on neurite growth of DRG neurons, and whether it promoted axonal regeneration, repaired DNA damage, and enhanced mitochondrial recovery of DRG neurons after cisplatin injury. Furthermore, we explored the possible interaction between NGF and TNF-α. Finally, we explored potential mechanisms mediated by the TNF-α receptor. This study is expected to provide a new strategy for targeted therapy of CIPN.

## 2. Materials and Methods

### 2.1. Animals

SPF-grade adult C57BL/6 mice (12–16 weeks old) were purchased from Chongqing Tengxin Biotechnology Co., Ltd. (Chongqing, China). Five or fewer mice were housed per cage under a 12/12 h light/dark cycle. Temperature was maintained at 20 °C–24 °C, with food and water available ad libitum. The study was conducted in accordance with ARRIVE guidelines. The requirements of the Ethics Committee of Guizhou University were followed in all animal experiments (grant no. EAE-GZU-2021-T082).

### 2.2. Culture and Treatment of DRG Neurons in Adult Mice

DRG neurons were isolated from C57BL/6 mice as described [22,23,24]. Briefly, ganglia tissue obtained from all spinal levels were placed in a cold dissection solution (130 mM NaCl, 5 mM KCl, 2 mM KH_2_PO_4_, 1.5 mM CaCl_2_, 6 mM MgCl_2_, 10 mM glucose, and 10 mM HEPES, pH 7.2). After tissue collection, all ganglia obtained were transferred to prewarmed dissection solution with 1 μg/mL collagenase A, 1 mg/mL trypsin and 50 μg/mL DNase I (Solarbio, Beijing, China) at 37 °C for 1 h [25]. The tissues were dissociated by grinding 20 times and filtered through a 70 μM filter, followed by centrifugation at 1200 rpm for 3 min. The pellet was resuspended in DMEM/F12 medium (BasalMedia, Shanghai, China) containing 10% fetal bovine serum (FBS) and 1% penicillin/streptomycin (both from Biosharp, Beijing, China). Cells were seeded onto 24-well plates pre-coated with 10 μg/mL laminin and 100 μg/mL poly-L-ornithine (both from Sigma-Aldrich, St. Louis, MO, USA) at a density of 3000 cells per well (the culture contained microglia and other cells, but only DRG neurons were counted), with or without pre-coated coverslips (same way as cell culture plate). The plates were then placed in a 37 °C incubator with 5% CO_2_ for culturing.

Cisplatin (Merck, HE, Darmstadt, Germany) was used at 12 µM to induce neural injury. This dosage impaired the DRG neurons and facilitated the study of molecular, metabolic, and structural alterations while minimizing substantial neuronal cell loss. After 24 h of treatment, cisplatin was removed by replacing with fresh medium. The cytokines were prepared from 10 μg/mL stock solutions made in PBS; different concentrations of the cytokines were added and cells were cultured for another 48 h.

### 2.3. Immunofluorescence Staining and Image Collection

DRG neurons were seeded onto glass cell culture cover slips for immunofluorescence staining. For these experiments, cells were fixed in 4% paraformaldehyde (Biosharp, Beijing, China), permeabilized with PBS solution containing 0.1% Triton X-100 (Solarbio, Beijing, China), and blocked using 3% bovine serum albumin (BSA) (Biofroxx, SN, Einhausen, Germany) in PBS. The primary antibodies used were rabbit anti-NF200 (Neurofilament 200) (1:50,000, Merck, HE, Germany), mouse anti-phospho-histone H2A.X (Ser139) (1:1500, Merck, HE, Germany), mouse anti-phospho-ATM (Ser1981) (1:10,000, Cell Signaling Technology, Danvers, MA, USA), mouse anti-MTCO1 (mitochondrial cytochrome c oxidase 1) (1:600, Invitrogen, Carlsbad, CA, USA), and mouse anti-phospho-NF-κB p65 (Ser536) (1:2000, Cell Signaling Technology, MA, USA). After washing three times with PBS containing 0.1% Tween (PBST), the slides were incubated with secondary antibodies (goat anti-mouse 488, or goat anti-rabbit cy5 both at 1:2000, Invitrogen, CA, USA) for 30 min. Following another three washes with PBST, the slides were mounted with ProLong™ Gold Antifade Mountant with DAPI (Invitrogen, CA, USA) and imaged using an Olympus fluorescence microscope (IX73, OLYMPUS cellSens Standard 4.1.1) using a 40× objective lens. To image the distribution of mitochondria in neurites, cells were observed and photographed under an Evident SpinSR10 super-resolution spinning disk confocal fluorescence microscope (Olyvia-3.4) using a 100× oil objective lens to collect image.

### 2.4. Neurite Length Measurement

To quantify the length of DRG neurite extension, staining with NF200, a neuron-specific cytoskeletal protein, was used to visualize the cell bodies and neurites of DRG neurons. Neurites were traced using the Segmented Lines tool in NIH ImageJ software (ImageJ 1.54f), and their lengths are analyzed. For each experimental condition, at least 10 randomly selected DRG neurons were analyzed. For each multiple-neurite-bearing DRG cell, 3–4 neurites were tracked, and the longest length from the cell body to the tip of the neurite was measured. The average length of these neurites was calculated. Three independent biological replicates were conducted. Data are presented as mean ± SEM, derived from more than 50 neurites analyzed per experimental condition.

### 2.5. Intensity Analysis of Immunofluorescence Staining

The fluorescence intensity of individual DRG neurons was measured using ImageJ software (ImageJ 1.54f). Cell bodies or neurites were selected using the Free Hand tool and the average intensity was measured. For each treatment condition, the immunofluorescence intensity from at least 15 DRG neurons was analyzed. Three independent biological replicates were performed, and the mean ± SEM values were calculated for at least 45 DRG neurons in each case.

### 2.6. RNA Interference

All small interfering RNA (siRNA) was synthesized by Genepharma (Shanghai, China). The siRNA sequence designs are shown in Table 1. According to the instructions of the reagent, siRNA was transfected into DRG neurons using RNAiMAX reagent (Invitrogen, CA, USA). After 24 h pretreatment with siRNA in neuronal basal medium, the siRNA containing medium was removed and replaced with full medium. Cells were then treated with 12 µM cisplatin for 24 h, followed by removal of cisplatin and co-culturing with NGF or TNF-α for an additional 48 h.

### 2.7. Real-Time Quantitative Polymerase Chain Reaction

Total RNA was extracted from DRG neurons by RNeasy Plus Micro Kit (QIAGEN, HE, Germany). Total RNA was reverse transcribed into cDNA using an Evo M-MLV reverse transcription premix kit (Accurate Biology, Changsha, China). Real-time quantitative polymerase chain reaction (RT-qPCR) was performed with SYBR Green Pro Taq HS premix qPCR kit (Accurate Biology, Changsha, China) and primers. The internal housekeeping gene used was Beta-2-Microglobulin (B2M), and relative mRNA quantities were calculated using the 2^−ΔΔCt^ method [26]. All primers were synthesized by Sangon (Shanghai, China). The forward and reverse sequences for each set of gene-specific primers are shown in Table 2.

### 2.8. Statistical Analysis

The data are presented as the mean ± SEM obtained from 3–5 independent biological replicates. One-way analysis of variance (ANOVA) was used to compare the differences among group means. A *p* value of less than 0.05 was considered statistically significant. Statistical analysis and graphing were performed using GraphPad Prism 9.5 software.

## 3. Results

### 3.1. TNF-α Promotes Neurite Outgrowth in Cultured DRG Neurons In Vitro

**Figure 1:**
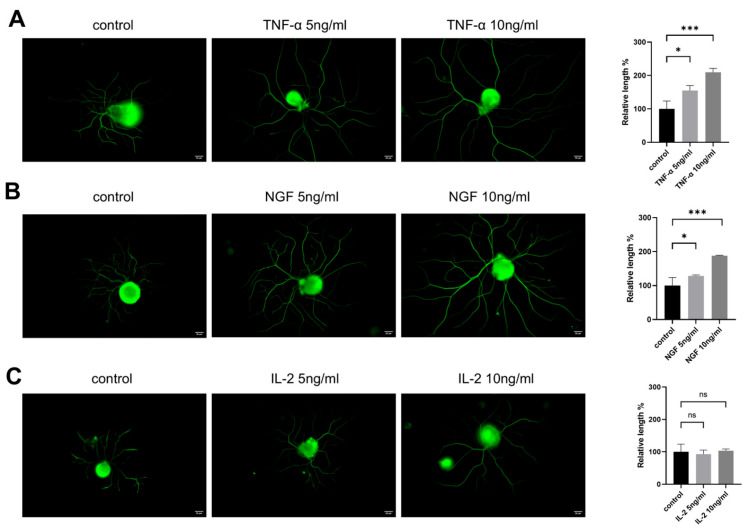
TNF-α promotes neurite outgrowth in cultured DRG neurons in vitro. DRG neurons were treated with different concentrations of TNF-α/NGF/IL-2 for 24 h. Cells were visible by immunofluorescence staining of NF200 (green). Scale bar = 20 μM. The neurite length of the DRG neuron was quantified. (**A**) DRG neuron cultured in normal medium with or without TNF-α; (**B**) DRG neuron cultured in normal medium with or without NGF; (**C**) DRG neuron cultured in normal medium with or without IL-2. Data are presented as mean ± SEM (*n* = 3 per group). ns: not significant, * *p* < 0.05, *** *p* < 0.001.

To evaluate the effects of cytokines on DRG neurons’ neurite outgrowth, neurite lengths from control groups and groups treated with different concentrations of cytokines were measured. As shown in Figure 1, DRG neurons treated with both NGF and TNF-α promoted neurite outgrowth in DRG neurons (Figure 1A,B), while IL-2, another important cytokine, had no significant effect on neurite growth (Figure 1C) after 24 h treatment.

### 3.2. TNF-α Promotes Neurite Regeneration in DRG Neurons Damaged by Cisplatin

**Figure 2:**
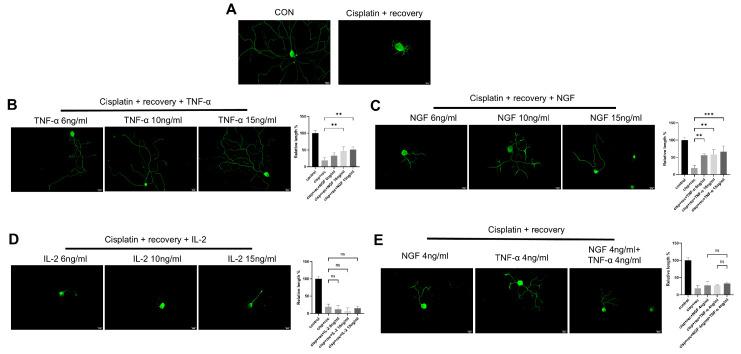
TNF-α promotes neurite regeneration in DRG neurons after cisplatin injury. DRG neurons received 12 μM cisplatin treatment for 24 h and then received fresh medium supplemented with different concentrations of TNF-α/NGF/IL-2 for 48 h. Cell bodies and neurites were visualized via immunofluorescence staining with NF200 (green). Scale bar = 20 μM. The neurite length of DRG neurons in each group was quantified. (**A**) DRG neuron with or without cisplatin injury; (**B**) DRG neuron cultured in NGF containing medium for 48 h after cisplatin injury; (**C**) DRG neuron cultured in TNF-α containing medium for 48 h after cisplatin injury; (**D**) DRG neuron cultured in IL-2 containing medium for 48 h after cisplatin injury; (**E**) DRG neuron cultured in medium containing both NGF and TNF-α for 48 h after cisplatin injury. Data are presented as mean ± SEM (*n* = 3 per group), ns: not significant, ** *p* < 0.01, *** *p* < 0.001.

To investigate the effect of TNF-α on neurite regeneration in DRG neurons damaged by cisplatin, we used different concentrations of TNF-α on DRG neurons after cisplatin-induced injury. Immunofluorescence staining revealed that TNF-α significantly promoted neurite regeneration in a dose-dependent manner (Figure 2B), with an effect similar to that of NGF (Figure 2C). Parallel experiments using IL-2 showed that IL-2 did not promote neurite regeneration in DRG neurons after cisplatin injury (Figure 2D). We further tested the combination of NGF and TNF-α, and as shown in Figure 2E; this combination did not show an additive or synergistic effect on neurite regeneration in DRG neurons after injury.

**Figure 3:**
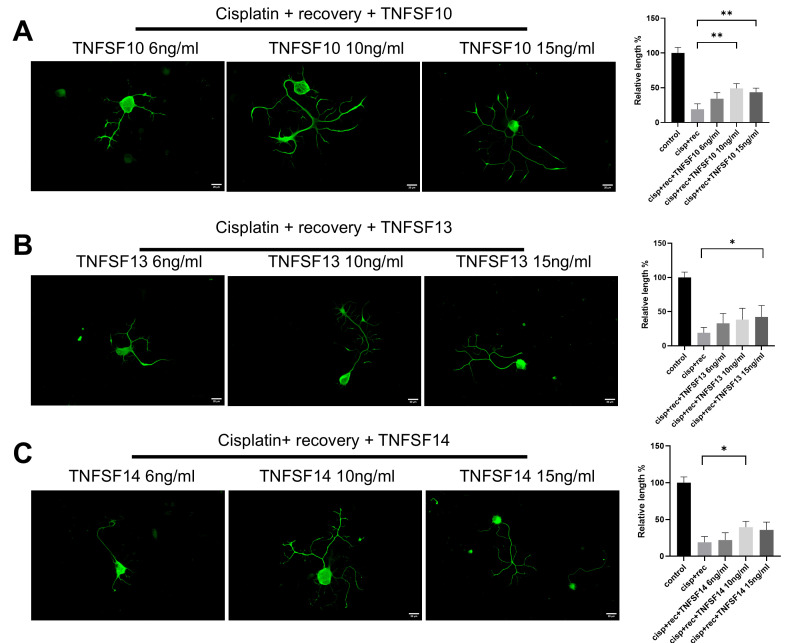
Members of TNF superfamily promote neurite regeneration in DRG neurons after cisplatin injury. DRG neurons received 12 μM cisplatin treatment for 24 h and then received fresh medium supplemented with different concentrations of TNFSF10/TNFSF13/TNFSF14 for 48 h. Cell bodies and neurites were visualized via immunofluorescence staining with NF200 (green). Scale bar = 20 μM. The neurite length of DRG neurons in each group was quantified. (**A**) DRG neuron cultured in TNFSF10 containing medium for 48 h after cisplatin injury; (**B**) DRG neuron cultured in TNFSF13 containing medium for 48 h after cisplatin injury; (**C**) DRG neuron cultured in TNFSF14 containing medium for 48 h after cisplatin injury. Data are presented as mean ± SEM (*n* = 3 per group), * *p* < 0.05, ** *p* < 0.01.

TNF-α is a classical member of the tumor necrosis factor superfamily (TNFSF). In order to further clarify the regulatory effect of TNF-α on neurite regeneration of injured DRG neurons and reveal its mechanism, we used members of the family, including TNFSF10, TNFSF13, and TNFSF14, for parallel experiments. As shown in Figure 3, all these TNF superfamily members promoted the regeneration of neurites in injured DRG neurons.

### 3.3. TNF-α Accelerates the Repairing Process of DNA Damage of DRG Neurons Induced by Cisplatin

**Figure 4:**
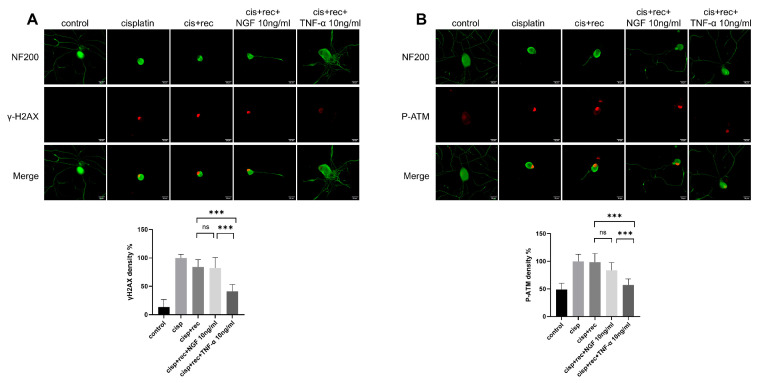
TNF-α accelerates the removing DNA damage in DRG neurons. DRG neurons received 12 μM cisplatin treatment for 24 h and then received fresh medium supplemented with different concentrations of TNF-α/NGF for 48 h. Cell bodies and neurites were visualized via immunofluorescence staining with NF200 (green). Scale bar = 20 μM. The intensity of staining was quantified for each group. (**A**) DNA damage in DRG neurons indicated by γH2AX (red) staining. In the cisplatin group, neurons were fixed direct after cisplatin treatment; in all recovery groups, neurons received 48 h recovery after cisplatin treatment. (**B**) DNA damage in DRG neurons indicated by P-ATM (red). Treatments were the same as (**A**). Data are presented as mean ± SEM (*n* = 3 per group), ns: not significant, *** *p* < 0.001.

ATM (ataxia telagiectasia mutated kinase) is an important gene in DNA damage repair pathways. When DNA double-strand break occurs, it undergoes trans-autophosphorylation at its Ser 1981 site, thus converting to monomer form and become activated [27], which then phosphorylates downstream target proteins, triggering DNA repair processes and activating H2AX [28], γH2AX serves as the initial signal molecule of DNA double-strand breaks. Therefore, phosphorylated γH2AX and ATM are used as sensitive markers to monitor DNA damage or DNA repair activity in many studies [25,29,30]. The data indicate that the intensity of γH2AX was markedly increase in the DRG neuron nuclei treated with cisplatin (Figure 4A). After the removal of cisplatin, the staining intensity of γH2AX in natural recovery group remained at almost the same level as in the cells just after cisplatin treatment. Even with NGF treatment, the staining intensity of γH2AX still remained at high level. However, in the TNF-α treatment group, the intensity of γH2AX was decreased significantly. Similar results were found with P-ATM staining (Figure 4B). These results indicated that after TNF-α treatment, DNA damage induced by cisplatin in DRG neurons was effectively removed, while NGF treatment showed no such effect.

### 3.4. TNF-α Promotes Regeneration of Mitochondrial in DRG Neurons

**Figure 5:**
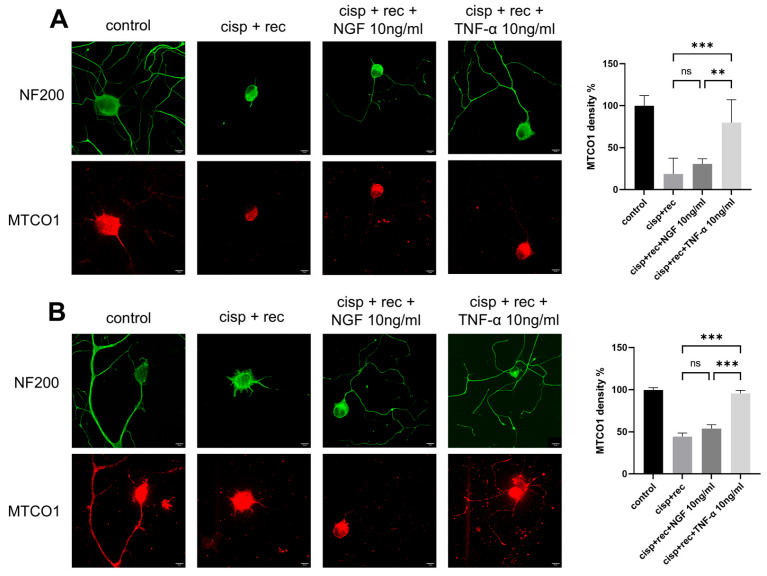
TNF-α promotes the regeneration of mitochondria in DRG neurons. DRG neurons received 12 μM cisplatin treatment for 24 h and then received fresh medium supplemented with different concentrations of TNF-α/NGF for 48 h. Cell bodies and neurites were visualized via immunofluorescence staining with NF200 (green), and mitochondria in DRG neurons are indicated by MTCO1 (red) staining. Scale bar = 20 μM. The intensity of staining was quantified for each group. (**A**) MTCO1 images in these groups were taken using a shorter exposure time to show the cell body clearly. (**B**) MTCO1 images in these groups were taken using a longer exposure time to show the neurites clearly. Data are presented as mean ± SEM (*n* = 3 per group), ns: not significant, ** *p* < 0.01, *** *p* < 0.001.

Cisplatin can cause severe mitochondrial damage in DRG neurons. We analyzed the effect of TNF-α on mitochondrial DRG neurons after cisplatin treatment and compared it with NGF. MTCO1 is a specific protein located in the inner membrane of mitochondria, and its content can be used to represent the amount of mitochondria. We used MTCO1 antibody for immunofluorescence imaging to represent the mitochondria in DRG neurons. As shown in Figure 5, strong MTCO1 staining was observed both in the cell bodies and the neurites of control neurons, which was significantly decreased after cisplatin treatment. After the removal of cisplatin, the staining intensity of MTCO1 indicated only very limited recovery under the conditions of natural recovery and NGF treatment. With TNF-α treatment, the MTCO1 staining intensity in the cell bodies of DRG neurons was significantly stronger than in the recovery group (Figure 5A).

For DRG neurons, regenerated neurites should have enough mitochondria inside to perform their corresponding functions. We further examined MTCO1 staining intensity in the regenerated neurites of DRG neurons. Although NGF group showed significant neurites regeneration, the numbers of mitochondria in the neurites were many fewer than in the TNF-α group, suggesting that TNF-α promoted the distribution of mitochondria into the neurites (Figure 5B).

### 3.5. The Effect of TNF-α on DRG Neurons Is Independent of the NGF Signaling Pathway

**Figure 6:**
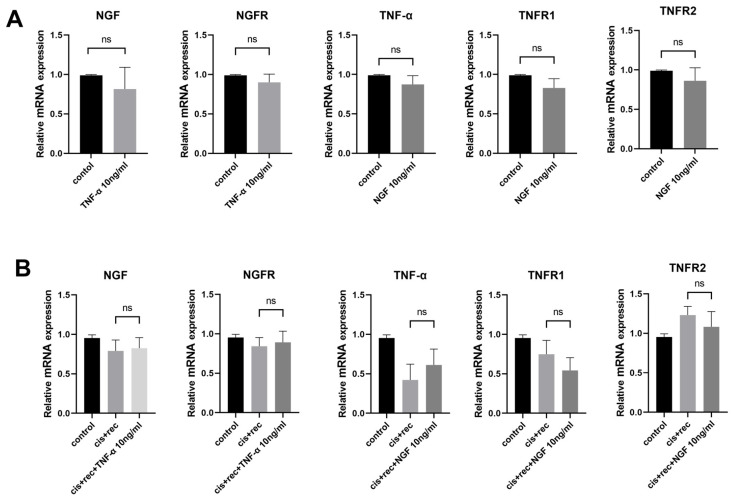
Effects of NGF and TNF-α on associated gene expression. (**A**) RT-qPCR was used to analyze the expression of related genes in DRG neurons after NGF or TNF-α treatment; (**B**) RT-qPCR analysis of the expression of related genes in DRG neurons treated with NGF or TNF-α after cisplatin injury. Data are presented as mean ± SEM (*n* = 3 per group), ns: not significant.

In order to elucidate the possible interaction between NGF and TNF-α signaling, we used the RT-qPCR method to examine expression of NGF, NGFR, TNF-α, TNFR1, TNFR2. The results from Figure 6 show that NGF had no effect on the expression of TNF-α and TNF-α receptors (TNFR1 and TNFR2) in DRG neurons. Similarly, TNF-α treatment did not alter the expression of NGF or NGFR (Figure 6A). Furthermore, we tested cisplatin-injured DRG neurons with NGF and with TNF-α. Compared with the Recovery group, the addition of NGF did not affect the expression of TNF-α, TNFR1, or TNFR2, and the addition of TNF-α did not affect the expression of NGF and NGFR (Figure 6B).

**Figure 7:**
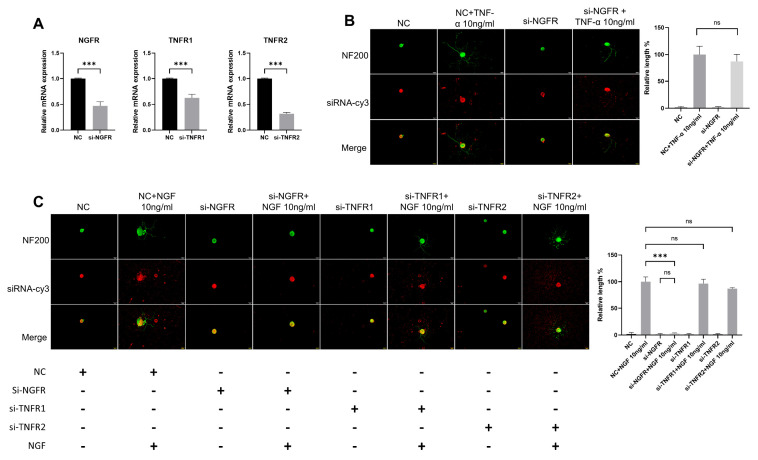
The effects of TNF-α on DRG neurons are independent of the NGF signaling pathway. The neurite length of DRG neurons in the control group and the experimental group was measured following siRNA transfection and TNF-α/NGF for 48 h. Cell bodies and neurites were visualized via immunofluorescence staining with NF200 (green) and siRNA was labeled with cy3 (red). Scale bar = 20 μM. (**A**) Knockdown efficiency of siRNA was determined by RT-qPCR; (**B**) NC group received nonspecific siRNA, NC+TNF-α group received nonspecific siRNA and TNF-α, si-NGFR group received NGFR siRNA, si-NGFR+TNF-α group received NGFR siRNA and TNF-α; (**C**) Treatments were performed as indicated. Data are presented as mean ± SEM (*n* = 3 per group), ns: not significant, *** *p* < 0.001.

To determine whether the observed effects were mediated by pathway crosstalk, we analyzed neuronal function following genetic knockdown of NGF or TNF-α signaling. We used siRNA to knockdown TNF-α receptors (TNFR1/TNFR2) or NGF receptor (NGFR), then applied NGF or TNF-α to DRG neurons and measured their neurite length. Three days post-transfection, all NGFR, TNFR1, and TNFR2 siRNA significantly reduced the targeted genes’ expression in DRG neurons (Figure 7A). The NF200 staining showed that NGF could still promote neurite growth in the si-TNFR1 group and the si-TNFR2 group, while it had almost no effect in si-NGFR group (Figure 7C). Meanwhile, TNF-α also played a normal role in promoting neurite growth of DRG neurons in the si-NGFR group (Figure 7B).

### 3.6. TNF-α Acts on DRG Neurons Depend on TNFR2-Mediated Signals

**Figure 8:**
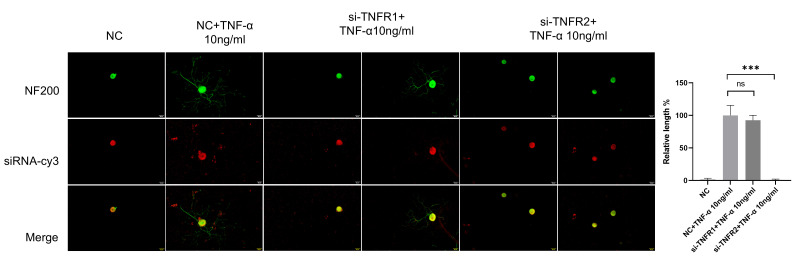
TNF-α acts on DRG neurons mainly through TNFR2-mediated signals. The neurite length of DRG neurons in the control group and the experimental group was measured following siRNA transfection and TNF-α for 48 h. Cell bodies and neurites were visualized via immunofluorescence staining with NF200 (green) and siRNA was labeled with cy3 (red). Scale bar = 20 μM. NC group received nonspecific siRNA, NC + TNF-α group received nonspecific siRNA and TNF-α, si-TNFR1 group received TNFR1 siRNA; si-TNFR1 + TNF-α group received TNFR1 siRNA and TNF-α; si-TNFR2 group received TNFR2 siRNA; si-TNFR2 + TNF-α group received TNFR2 siRNA and TNF-α. Data are presented as mean ± SEM (*n* = 3 per group), ns: not significant, *** *p* < 0.001.

TNF-α acts through two receptors with different structures (TNFR1 and TNFR2), and there are significant differences in expression patterns and functions between these. TNFR1 and TNFR2 are relatively abundantly expressed in DRG neurons. At present, it is not clear which receptor is the main functioning receptor in DRG neurons. Thus, we tested TNFR1 and TNFR2 siRNA on DRG neurons. The results of the NF200 staining showed that DRG neurons in the si-TNFR1 group showed normal neurite outgrowth, while in the si-TNFR2 group, neurite outgrowth was totally inhibited (Figure 8).

**Figure 9:**
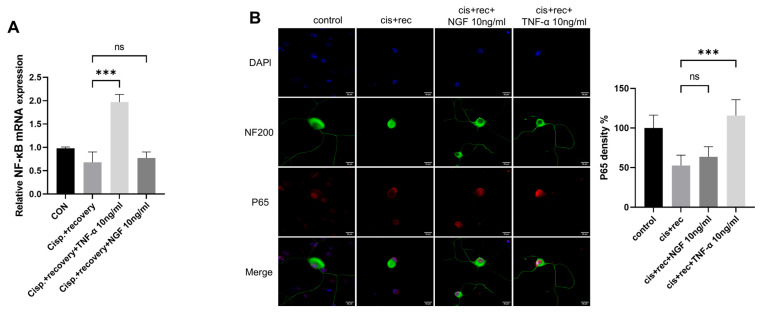
Downstream signal of TNFR2 in DRG neurons was activated by TNF-α. DRG neurons received 12μM cisplatin treatment for 24 h and then received fresh medium supplemented with different concentrations of TNF-α/NGF for 48 h. (**A**) Expression of NF-κB in DRG neurons was determined by RT-qPCR analysis; (**B**) P65 nuclear translocation was determined in DRG neuron, Cell bodies and neurites were visualized via immunofluorescence staining with NF200 (green), P65 indicated by P65 (red) staining and nucleus indicated by DAPI (blue). Scale bar = 20 μM. Data are presented as mean ± SEM (*n* = 3 per group), ns: not significant, *** *p* < 0.001.

TNFR2 was found to be the main functioning receptor of TNF-α in DRG neurons. We further explored the downstream signaling molecules of TNFR2 in DGR neurons, including NF-κB and P65 [31,32]. We examined the expression of NF-κB; the RT-qPCR results showed that TNF-α upregulated NF-κB in DRG neurons, while NGF had no such effect (Figure 9A). P65 immunofluorescence staining revealed that cisplatin treatment weakened the nuclear compartment of p65 in the nuclei of DRG neurons. The treatment with TNF-α restored the nuclear compartment of p65 in DRG neurons, while NGF did not have such effect (Figure 9B).

## 4. Discussion

In this study, we explored the function of TNF-α as a potential therapeutic molecule against CIPN. CIPN seriously restricts the clinical application of chemotherapy drugs including cisplatin, and the symptoms are persistent, with no effective treatment. Despite extensive investigation into NGF’s neurorestorative functions, its effects are controversial and may even aggravate neuronal damage under some pathological conditions [33,34]. In recent years, TNF-α has attracted wide attention because of its dual role in nerve regeneration. On one hand, TNF-α participates in neuropathic pain as a pro-inflammatory factor [35,36]; on the other hand, it may promote axon outgrowth in normal culture conditions [14,17]. However, the specific role of TNF-α in chemotherapy drug induced DRG neuron injury and its potential synergistic effect with NGF are not clear. In this study, we found that TNF-α promoted neurite regeneration of DRG neurons after cisplatin treatment. The experimental results indicate that TNF-α has significant neurorestorative effects. However, when DRG neurons were treated with the classic pro-inflammatory cytokine IL-2, this showed no such effect. This finding indicates that only certain cytokines have the ability to help repairing damaged neurons. Notably, by systematically comparing the effects of TNF-α with those of other members of the TNFSF family (TNFSF10/13/14), it was found that although these members have weaker regenerative capabilities than TNF-α, they all exhibited a certain degree of neurite regeneration-promoting effects. This result suggests that promoting neurite regeneration may be a common biological characteristic of the TNFSF family. In contrast, TNF-α demonstrated more significant neurorestorative effects, which may be attributed to its more efficient receptor-signaling system and specific interactions with the neuronal microenvironment. This unique signaling mechanism gives TNF-α a clear advantage in promoting neuronal repair.

When the DRG is injured, macrophage infiltration is a common phenomenon. There are many reports indicating that these infiltrated macrophages can secrete TNF-α [37,38], and microglia in DRG can also secrete TNF-α after stimulation. The effects of TNF-α on DRG neurons have not been clearly determined, and one of the important reasons is the diversity of TNF-α signals. TNF-α can act on both neurons and supporting cells such as microglia around neurons, and these supporting cells may secrete neurotrophic factors such as NGF under the stimulation of TNF-α, which makes the effect of TNF-α on DRG neurons even more complicated [18,19,20,21]. Therefore, we need to be clear about the potential interaction of neurotrophic factors, represented by NGF, with TNF-α in DRG neurons before researching the mechanism.

Our first attempt was to determine whether NGF can work synergistically with TNF-α. Our results showed that the combined treatment with TNF-α and NGF had no synergistic or additive effect, a finding inconsistent with the hypothesis that TNF-α affects DRG neurons by inducing NGF expression.. However, if we assume that TNF-α and NGF affect DRG neurons through separate signal pathways without interaction, this is the expected result. We then detected the expression of NGF and NGFR, the key molecules in NGF signaling, in the DRG treated with TNF-α. The results showed that the expression of these two factors did not increase after the treatment of TNF-α. Similarly, in the DRG neurons treated with NGF, the key molecules in TNF-α signaling, i.e., TNF-α itself, TNFR1, and TNFR2, did not change at all. This is also in line with our hypothesis. Furthermore, when we directly used siRNA to knock down NGFR and inhibited NGF signaling almost totally, the effect of TNF-α on DRG neurons was almost completely unaffected. Meanwhile, our results showed that TNF-α had some significantly better effects than NGF in promoting the repair of DRG neurons from cisplatin-induced injury, including accelerating the removal of DNA damage and promoting the recovery of mitochondrial level. As these effects could not be seen in the NGF-treated DRG neurons, this hints that some signals other than NGF signals must have been involved in the repair of DRG neurons after TNF treatment. Based on the above results, we believe that there is sufficient evidence to show that the effect of TNF-α on DRG neurons is accomplished independent of this NGF-signaling pathway.

Of course, this experiment focused only on NGF and could not rule out the possibility that TNF-α can promote injury repair by inducing other neurotrophic factors. Therefore, we further tested the dependence of TNF-α on its own receptors in DRG neurons. The results show that when TNFR2 was knocked down, the effect of TNF-α was almost completely blocked. This result indicates that even if there are other neurotrophic factors that may be involved, these are downstream events after the activation of TNFR2.

Cisplatin can cause very broad DNA damage in DRG neurons, while the speed of DNA repair in DRG neurons is relatively limited [25,30]. Thus, in the nuclei of DRG neurons two days after cisplatin treatment, there was still plenty of γH2AX and P-ATM staining. In this study, we also found that NGF treatment of cisplatin-injured DRG neurons had little effect on removing DNA damage. However, when using TNF-α to treat DRG neurons, the DNA repair speed was greatly accelerated, resulting in much weaker staining with both γH2AX and P-ATM.

Mitochondrial damage caused by cisplatin is another important sign of neuron injury. The recovery of mitochondrial numbers is an important factor in the functional recovery of neurons. In our results, the intensity of MTCO1 staining showed that the treatment with TNF-α after injury promoted the regeneration of mitochondria. Also, after cisplatin injury, mitochondria in the regenerated neurites of DRG neurons remained at a very low level in the NGF group, which indicates that these regenerated neurites were still not in normal functional mode. After TNF-α treatment, the amount of mitochondria in the neurite significantly increased, suggesting that there were many more mitochondria in these regenerated neurites, which hints these regenerated neurites may function better. As we know, mitochondrial damage can be induced not only by cisplatin, but also by many other chemotherapy drugs, such as vinblastine [39], bortezomib [40,41], and paclitaxel [42]. The ability of TNF-α to promote mitochondrial regeneration may also help in recovery of nerve damage caused by these drugs, which is worth further exploration.

TNF-α can trigger very different biological functions according to which receptor, TNFR1 or TNFR2, has been activated [43]. Beyond its pro-inflammatory functions, TNFR1 possesses a cytoplasmic death domain capable of initiating both apoptotic and necroptotic pathways. These cell death mechanisms underlie TNF-α’s tumoricidal activity [44,45]. In contrast, TNFR2 mediates very complicated signaling and is capable of inducing anti-inflammatory responses while promoting neurorestorative effects and remyelination [46]. As the two receptors show quite different functions, we aimed to elucidate which receptor is the primary one. In this study, we found that both receptors were expressed in DRG neurons, but TNFR1 siRNA did not affect TNF-α’s ability, while TNFR2 siRNA was able to abolish TNF-α’s ability, indicating that TNFR2 is the main receptor for TNF-α in DRG neurons.

Notably, previous studies have demonstrated that TNF-α contributes to bortezomib-induced CIPN [36]. These findings further highlight the dual role of TNF-α in nerve regeneration and the complexity of its effects in DRG neurons. This work indicates that JNK activation by TNF-α is mediated by both TNFR1 and TNFR2, yet even in TNFR2-knockout mice, JNK activation can still be observed. In our experiments, siRNA-mediated knockdown of TNFR2 completely abolished TNF-α’s promotive effect on neurite outgrowth. Based on these results, we hypothesize that the TNFR2-dependent NF-κB signaling pathway might be the main pathway contributing to TNF-α’s functions, such as neurite regeneration, removal of DNA damage, and mitochondrial regeneration.

Previous studies have shown that TNFR2 can activate both the canonical and non-canonical NF-κB pathways [47,48,49], In this experiment, we observed that TNF-α promoted the nuclear translocation of p65 and upregulated the expression of NF-κB in DRG neurons, suggesting that TNF-α indeed acted through TNFR2 and activated the canonical TNF-α signaling pathway in the DRG neurons. However, it is not clear yet which molecule is the key factor for promoting the neurite outgrowth of DRG neurons through this signaling pathway; we will keep working on this. In addition, whether the ability of TNF-α to accelerate DNA damage removal and mitochondrial regeneration in injured DRG neurons relates to the activation of NF-κB or not requires further investigation. Since TNF-α may also activate other signaling pathways (such as PI3K/Akt and MAPK pathways) via TNFR2, it is possible that these pathways may also contribute to the recovery of neurons. However, we have not studied whether these pathways also play a role in DRG neurons and whether there are interactions between these pathways, and further investigation is still needed to determine which pathways are involved in the recovery process. We will employ single-cell sequencing technology and other methods to further analyze the transcriptomic changes in DRG neurons under the action of TNF-α, which may reveal more involved targets and help us to identify the link between TNF-α and its effects.

While our data indicate that TNF-α directly activates neuronal TNFR2 to promote recovery processes, two limitations remain. First, siRNA-mediated TNFR2 knockdown requires additional validation at the protein level and assessment of compensatory TNFR1 signaling. Second, the presence of non-neuronal cells in DRG neuron cultures opens the possibility of indirect effects; however, the efficacy of TNFR2 knockdown strongly argues against glia-driven mechanisms. Importantly, given the fact that mitochondrial damage and DNA repair deficits are common neurotoxic mechanisms shared by multiple chemotherapy agents (e.g., paclitaxel, bortezomib, and vinblastine) [41,42,43,44], our findings suggest the potential applicability of TNF-α/TNFR2 signaling in broader CIPN contexts.

Our study demonstrates that TNF-α promotes the recovery of cisplatin-injured DRG neurons through a TNFR2/NF-κB-dependent pathway in vitro. However, to fully assess the translational potential of these findings, future research must extend into in vivo models of CIPN. The complex interplay of systemic drug distribution, neuro-immune interactions, and behavioral manifestations of neuropathy such as sensory deficits, pain hypersensitivity, and motor dysfunction might be adequately evaluated only in a living organism.

Moving forward, we will establish a rodent CIPN model and further study the efficacy and safety of TNF-α or TNFR2-specific agonists. Behavioral assessments, including mechanical allodynia and thermal hyperalgesia, will be combined with histopathological analyses of DRG integrity, axonal regeneration, and mitochondrial recovery to determine the efficiencies of treatments. Given TNF-α’s multiple roles in neuroprotection and inflammation, selective TNFR2 activation may offer a more targeted approach, maximizing regenerative benefits while minimizing pro-inflammatory side effects.

Additionally, mechanistic studies using transcriptomic and proteomic profiling will further elucidate downstream NF-κB-regulated genes and proteins responsible for neurite outgrowth, DNA repair, and mitochondrial biogenesis. These insights may uncover novel therapeutic targets and refine our understanding of TNFR2-mediated neurorestoration. Together, these future directions will bridge the gap between in vitro findings and clinical application, advancing TNF-α/TNFR2 signaling as a viable strategy for CIPN treatment.

## 5. Conclusions

In summary, our findings demonstrate TNF-α’s crucial role in mitigating cisplatin-induced neurotoxicity via the TNFR2/NF-κB pathway, suggesting a potential therapeutic strategy for CIPN intervention. Future translational research should employ multidisciplinary approaches to facilitate clinical translation of these findings.

## Figures and Tables

**Table 1:** siRNA sequence.

siRNA Name	Sequence (5′-3′)
si-NC	sense: 5′-UUCUCCGAACGUGUCACGUTT-3′
	anti-sense: 5′-ACGUGACACGUUCGGAGAATT-3′
si-NGFR	sense: 5′-GGGCCUUGUGGCCUAUAUUTT-3′
	anti-sense: 5′-AAUAUAGGCCACAAGGCCCTT-3′
si-TNFR1	sense: 5′-UACGGCUUCCCAGAAUUACTT-3′
	anti-sense: 5′-GUAAUUCUGGGAAGCCGUATT-3′
si-TNFR2	sense: 5′-CUGAUGAAAUCCCAGGATT-3′
	anti-sense: 5′-UCCUGGGAUUUCUCAUCAGTT-3′

NC: nonspecific control; NGFR: nerve growth factor receptor; TNFR1: tumor necrosis factor receptor 1; TNFR2: tumor necrosis factor receptor 2; siRNA: small interfering RNA.

**Table 2:** Forward and reverse sequences of each gene analyzed by RT-qPCR.

Primer Name	Sequence (5′-3′)
B2M	Forward: 5′-ATCCAAATGCTGAAGAACGG-3′
	Reverse: 5′-ATCAGTCTCAGTGGGGGTGA-3′
NF-κB	Forward: 5′-TGAAACACTGGAAGCACGGATGAC-3′
	Reverse: 5′-TCTCCTCCGCCTTCTGCTTGTAG-3′
NGF	Forward: 5′-AAGTGCCGAGCCTCCAATCC-3′
	Reverse: 5′-TTCTCATCTGTTGTCAACGCCTTG-3′
NGFR	Forward: 5′-CACAGCGACAGCGGCATCTC-3′
	Reverse: 5′-AGCAGCTTCTCGACCTCCTCAC-3′
TNF-α	Forward: 5′-GCCACCACGCTCTTCTGTCTAC-3′
	Reverse: 5′-GGTTTGTGAGTGTGAGGGTCTGG-3′
TNFR1	Forward: 5′-AGTTCCAACGCTACCTGAGTGAG-3′
	Reverse: 5′-ACGGTGTTCTGAGTCTCCTTACAG-3′
TNFR2	Forward: 5′-CCTGCCTACAAAGAGATGCCAAG-3′
	Reverse: 5′-GAACTGGGTGCTGTGGTCAAC-3′

## Data Availability

Data will be made available on request.
